# Rational Choice of Antibiotics and Media for *Mycobacterium avium* Complex Drug Susceptibility Testing

**DOI:** 10.3389/fmicb.2020.00081

**Published:** 2020-02-19

**Authors:** Jérémy Jaffré, Alexandra Aubry, Thomas Maitre, Florence Morel, Florence Brossier, Jérôme Robert, Wladimir Sougakoff, Nicolas Veziris, the CNR-MyRMA (Centre National de Référence des Mycobactéries et de la Résistance des Mycobactéries aux Antituberculeux)

**Affiliations:** ^1^AP-HP (Assistance Publique Hôpitaux de Paris), Centre National de Référence des Mycobactéries et de la Résistance des Mycobactéries aux Antituberculeux, Groupe Hospitalier Universitaire Sorbonne Université, Hôpital Pitié Salpêtrière, Paris, France; ^2^INSERM, U1135, Centre d’Immunologie et des Maladies Infectieuses, Sorbonne Université, Cimi-Paris, Paris, France; ^3^AP-HP (Assistance Publique Hôpitaux de Paris), Groupe Hospitalier Universitaire, Sorbonne Université, Hôpital Saint-Antoine, Paris, France

**Keywords:** drug susceptibility testing, *Mycobacterium avium* complex, SLOMYCO Sensititre^TM^, 7H9, Mueller Hinton, clarithromycin, amikacin

## Abstract

The Clinical and Laboratory Standards Institute recommends the use of Mueller Hinton (MH) medium to perform drug susceptibility testing (DST) of *Mycobacterium avium* complex (MAC) using the microdilution method. For MAC, there has been no study on the impact of media on the determination of minimum inhibitory concentrations (MICs) of antibiotics other than clarithromycin. This study aimed at determining the impact of two media used for DST of MAC and at augmenting the number of pertinent MICs for MAC species encountered in clinical practice. MICs of antibiotics used for the treatment of MAC infections were determined for 158 clinical MAC isolates (80 *M. avium*, 40 *M. intracellulare*, 35 *M. chimaera*, two *M. yongonense* and one *M. timonense*) in MH and 7H9 broths using the SLOMYCO Sensititre^TM^ system (TREK Diagnostic Systems, East Grinstead, United Kingdom). The modal MICs determined in both media were the same for linezolid, moxifloxacin, rifabutin and amikacin but not for clarithromycin, rifampin and ethambutol. The kappa test for MICs converted to susceptibility categories showed an excellent agreement for clarithromycin, a moderate agreement for linezolid and a weak agreement for moxifloxacin and amikacin. For amikacin, 7H9 allowed a better distinction (fewer intermediate strains) of wild-type populations than MH. Existing breakpoints for linezolid and moxifloxacin are spread through the distribution of MICs for wild-type populations. The only breakpoints that can be used rationally are those for amikacin and clarithromycin. For amikacin, 7H9 performs better than MH, whereas both media perform equally for clarithromycin. Given that testing in 7H9, as opposed to MH, allows easier MIC measurements and yields greater reproducibility, we propose the use of 7H9 medium for DST of MAC.

## Introduction

Nontuberculous mycobacteria (NTM) are ubiquitous microorganisms isolated mainly from water and soil. In 1959, Runyon proposed the first classification of NTM into four groups, the first three comprising “Slowly Growing Mycobacteria” (SGM) and the fourth of “Rapidly Growing Mycobacteria.”

The most common SGM are the species belonging to the *Mycobacterium avium* complex (MAC), comprising especially *Mycobacterium avium*, *Mycobacterium intracellulare* and *Mycobacterium chimaera*. MAC organisms can cause different conditions in humans such as pulmonary disease, lymphadenitis and disseminated infection (Griffith et al., 2007). The incidence of MAC infections is increasing in most industrialized countries, possibly because of the increase in immunocompromised and/or older patients (Griffith et al., 2007; Prevots et al., 2010). These infections require antibiotic therapy based on macrolides (azithromycin or clarithromycin) combined with a rifamycin (rifampin or rifabutin) and ethambutol. Parenteral or inhaled amikacin may be added to this regimen for severe cases (Griffith et al., 2007, 2018; Olivier et al., 2017). Clofazimine, moxifloxacin, linezolid and bedaquiline are alternative drugs proposed mainly for the treatment of infections caused by macrolide-resistant MAC, but clinical evidence of their efficacy is lacking (Griffith et al., 2007; Koh et al., 2013; Jo et al., 2014; Philley et al., 2015).

The Clinical and Laboratory Standards Institute (CLSI) published guidelines for drug susceptibility testing (DST) of NTM in 2011, mainly reproduced by the British Thoracic Society (BTS) in 2017, and updated in 2018 (Clinical and Laboratory Standards Institute [CLSI], 2011, 2018; Haworth et al., 2017). As for MAC, both CLSI and BTS agree that, for clarithromycin susceptibility, the isolate be tested prior to the initiation of treatment in patients who satisfy the diagnostic criteria of nontuberculous mycobacterial lung disease of the American Thoracic Society/Infectious Disease Society of America. Indeed, a strong relationship between *in vitro* activity and *in vivo* efficacy of clarithromycin has been well established in clinical trials (Dautzenberg et al., 1995; Wallace et al., 1996, 2014; Tanaka et al., 1999; Kobashi et al., 2012). When testing macrolides, the pH of the medium is critical for the interpretation of the minimal inhibitory concentration (MIC) (Truffot-Pernot et al., 1991). Cation-adjusted Mueller Hinton (MH) medium has a pH of 7.4 whereas 7H9 medium has a pH of 6.8. Consequently, CLSI guidelines propose different breakpoints according to the medium employed only for clarithromycin (Clinical and Laboratory Standards Institute [CLSI], 2011, 2018).

The BTS also recommends amikacin susceptibility testing of isolates collected prior to initiation of treatment (Haworth et al., 2017). New CLSI guidelines published in 2018 likewise propose amikacin testing and give two breakpoints, depending on the route of administration (intravenous or inhaled) (Clinical and Laboratory Standards Institute [CLSI], 2018).

In case of resistance to clarithromycin, CLSI recommends DST of moxifloxacin and linezolid, whereas BTS recommends testing a wider panel of antibiotics to guide treatment regimens (Clinical and Laboratory Standards Institute [CLSI], 2011, 2018; Haworth et al., 2017).

Although used for the treatment of MAC infections, no clinical breakpoints have been defined for ethambutol and rifamycins.

Therefore, our goals were to determine the impact of the media used for MAC DST and to augment MIC data for clinically relevant MAC species.

## Materials and Methods

### Bacterial Strains and Growth Conditions

The study involved 158 clinically relevant MAC isolates sent to the French National Reference Centre for Mycobacteria from 2015 to 2017. The isolates were grown on Löwenstein-Jensen medium and incubated at 37°C in ambient air. Before April 2016, isolates were identified with the GenoType Mycobacterium CM line probe assay (Hain Lifescience, Nerhen, Germany) associated with the sequencing of the internal transcribed spacer (ITS) region and, after this date, with the GenoType NTM-DR assay (Hain Lifescience). Mixed NTM infections were ruled out from the study.

The reference strain *M. avium* ATCC 700898 was used for the quality control of MIC determinations and for reproducibility testing of the technique.

### MIC Determination

For each strain, DST was performed in parallel once in MH and once in 7H9 broth by microdilution using the SLOMYCO Sensititre^TM^ system (TREK Diagnostic Systems, East Grinstead, United Kingdom) and MICs were determined according to the instructions of the CLSI and manufacturer. In practice, a suspension (0.5 McFarland standard) of each isolate was transferred into the two assessed media: cation-adjusted MH broth and Middlebrook 7H9 broth, both supplemented with 5% oleic albumin dextrose catalase (OADC). The final suspension was inoculated into the tray that was subsequently incubated at 37°C in ambient air. The tray was examined after 7 days of incubation and MICs were determined visually using an inverted mirror. In case of insufficient growth, trays were reincubated and read again after 10 to 14 days. The lowest concentration of antimicrobial that inhibited visible growth was taken as the MIC. Results were considered to be invalid if no growth was detected in the control well.

For each strain, 13 antimicrobial agents were tested but only results concerning the MICs of those recommended for treatment of MAC infections (clarithromycin, moxifloxacin, linezolid, amikacin, ethambutol, rifampin, rifabutin) are presented in this work.

The range of tested concentrations for each antibiotic of interest is given in Supplementary Table S1.

### SIR (Susceptibility, Intermediate Susceptibility, Resistance) Categorization

For clarithromycin, two breakpoints were used to categorize MICs according to the medium inoculated: with MH broth (pH 7.4), clarithromycin susceptibility was assumed at an MIC of ≤8 mg/L as opposed to ≤16 mg/L when 7H9 broth (pH 6.8) was used. No breakpoint is proposed in the recent CLSI guidelines (Clinical and Laboratory Standards Institute [CLSI], 2018) to interpret the clarithromycin MIC measured in 7H9. We therefore used the breakpoint proposed in 2011 for the radiometric instrument method (Clinical and Laboratory Standards Institute [CLSI], 2011) since the pH is the same in both cases. To interpret moxifloxacin and linezolid MICs obtained in both media, we used the breakpoints defined by the CLSI for broth microdilution at pH 7.4 (susceptible strain if moxifloxacin MIC ≤ 1 mg/L and linezolid MIC ≤ 8 mg/L) (Clinical and Laboratory Standards Institute [CLSI], 2018). For amikacin, we applied the breakpoint defined by the CLSI in 2018 for the intravenous route (susceptible strain if MIC ≤ 16 mg/L) (Brown-Elliott et al., 2013; Clinical and Laboratory Standards Institute [CLSI], 2018). These breakpoints are summarized in Table 1.

**TABLE 1:** Antimicrobial agents and interpretive criteria for *Mycobacterium avium* complex used in the study.

**Antimicrobial agent**	**Medium**	**MIC (mg/L) for category**		
		**S**	**I**	**R**
Amikacin (IV)	MH and 7H9	≤16	32	≥64
Amikacin (liposomal or inhaled)	MH and 7H9	≤64	–	≥128
Clarithromycin	MH (pH 7.3–7.4)	≤8	16	≥32
	7H9 (pH 6.8)	≤16	32	≥64
Linezolid	MH and 7H9	≤8	16	≥32
Moxifloxacin	MH and 7H9	≤1	2	≥4

*S, susceptible; I: intermediate; R: resistant.*

### Statistical Analysis

Reproducibility was evaluated by measuring MICs of the reference strain *M. avium* ATCC 700898 six times in both media. The results are expressed as a percentage of agreement compared to reference values given by the CLSI.

MIC_50_ and MIC_90_ values were determined from MIC distributions and were defined as the MICs required to inhibit the growth of 50% and 90% of the studied strains.

ECOFF and modal MIC (defined by the most frequent MIC value for each distribution of aggregated MICs for each species and drug), were calculated using the EUCAST excel tool ECOFF Finder (European Committee on Antimicrobial Susceptibility Testing [EUCAST], 2019, http://www.eucast.org/mic_distributions_and_ecoffs/). We chose to present ECOFF 95% values since the MICs showed a non-wild-type distribution, some reflecting acquired resistance.

MIC and susceptibility results obtained with both media were compared. Confirmed discordant results were classified as either very major errors [VME, defined as resistance (R) in MH but susceptibility (S) in 7H9], major errors (ME, S in MH and R in 7H9), or minor errors [mE, intermediate (I) in one medium but S or R in the other]. MICs determined in MH broth were considered the reference values.

The agreement between both methods was expressed as percent concordance and the strength of the agreement was determined using kappa scores which are considered to be excellent, strong, moderate, weak, very weak or null when they are 1–0.8, 0.8–0.6, 0.6–0.4, 0.4–0.2, 0.2–0 or ≤0, respectively.

## Results

### Strains Included in the Study

A total of 158 MAC isolates were included in the study. These strains were collected from 141 patients. 14 patients had between two and four strains included in the study (total of 31 strains), with a mean interval between two strains of 6.5 months. These strains belonged to different species: 80 *M. avium* (51%), 40 *M. intracellulare* (25%), 35 *M. chimaera* (22%), 2 *Mycobacterium yongonense* (1%) and 1 *Mycobacterium timonense* (1%). These strains were isolated from various pulmonary specimens (90%) and extrapulmonary samples (10%) such as blood cultures and lymph node biopsies. They were isolated from 141 patients, 96 (68%) who had a history of long-term macrolide treatment, 20 (14%) who had no history of treatment, and 25 (18%) whose history of treatment was unknown.

### Reproducibility

Reproducibility results are shown in Supplementary Table S2.

The concordance percentages obtained for the three antibiotics for which the CLSI proposes breakpoints were 100% with both media tested, except for clarithromycin in MH (67%).

### MICs

**FIGURE 1:**
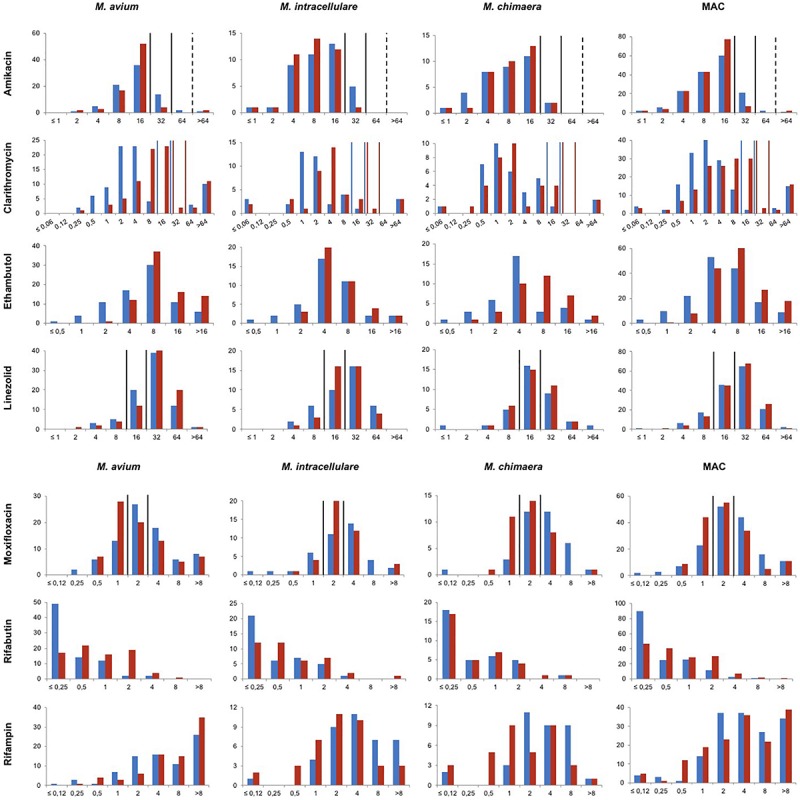
Distribution of MICs for *M. avium*, *M. intracellulare*, *M. chimaera* and *M. avium* complex clinical isolates determined in MH and 7H9 broths. *x*-axis represents the MICs in mg/L. *y*-axis represents the number of strains. Blue and red bars represent values obtained in MH and 7H9 broth, respectively. Vertical lines represent the CLSI susceptibility breakpoints. For amikacin, breakpoints for the intravenous route are represented in full lines and for the liposomal or inhalation route in dotted lines. For clarithromycin, breakpoints for MICs determined in MH and 7H9 are represented in blue and red lines, respectively.

The distribution of MIC values determined in MH and 7H9 for MAC species is shown in Figure 1 and in Supplementary Figures S1, S2. MIC_50_, MIC_90_, Modal MIC, percentage of concordance, kappa and ECOFF values are shown in Table 2.

**TABLE 2:** MICs (mg/L) determined in MH and 7H9 for *M. avium* complex overall and for the species *M. avium, M. intracellulare*, and *M. chimaera*.

						**MIC values**	
	**N**	**Modal MIC MH/7H9**	**MIC_50_ MH/7H9**	**MIC_90_ MH/7H9**	**Tentative ECOFF 95% MH/7H9**	**% Concordance**	**Kappa coefficient**
**Amikacin**							
*M. avium* complex	158	16/16	16/16	32/16	32/32	57	0.39
*M. avium*	80	16/16	16/16	32/16	32/32	56	0.3
*M. intracellulare*	40	16/8	8/8	32/16	NA/32	55	0.39
*M. chimaera*	35	16/16	8/8	16/16	32/32	57	0.43
**Clarithromycin**							
*M. avium* complex	158	2/8	2/8	64/>64	8/32	27	0.18
*M. avium*	80	2/16	3/8	>64/>64	8/32	19	0.12
*M. intracellulare*	40	1/4	2/4	8/16	8/16	20	0.13
*M. chimaera*	35	1/2	1/2	8/16	4/16	46	0.37
**Ethambutol**							
*M. avium* complex	158	4/8	4/8	16/>16	16/32	37	0.18
*M. avium*	80	8/8	8/8	16/>16	32/16	35	0.13
*M. intracellulare*	40	4/4	4/4	8/16	16/8	43	0.17
*M. chimaera*	35	4/8	4/8	16/16	8/32	34	0.17
**Linezolid**							
*M. avium* complex	158	32/32	32/32	64/64	64/64	60	0.43
*M. avium*	80	32/32	32/32	64/64	64/64	49	0.25
*M. intracellulare*	40	32/16	32/24	64/32	>64/64	70	0.58
*M. chimaera*	35	16/16	16/16	32/32	64/64	74	0.61
**Moxifloxacin**							
*M. avium* complex	158	2/2	2/2	8/8	8/4	46	0.24
*M. avium*	80	2/1	2/2	8/8	8/4	55	0.35
*M. intracellulare*	40	4/2	3/2	8/4	8/4	48	0.26
*M. chimaera*	35	2/2	4/2	8/4	8/4	20	−0.08
**Rifabutin**							
*M. avium* complex	158	≤0.25/≤0.25	≤0.25/0.5	2/2	1/0.5	39	0.17
*M. avium*	80	≤0.25/0.5	≤0.25/1	1/2	0.5/4	27	0.06
*M. intracellulare*	40	≤0.25/≤0.25	≤0.25/0,5	2/2	0.5/0.5	48	0.3
*M. chimaera*	35	≤0.25/≤0.25	≤0.25/0.5	2/2	0.5/0.5	57	0.37
**Rifampin**							
*M. avium* complex	158	2/>8	4/4	>8/>8	>8/NA	35	0.09
*M. avium*	80	>8/>8	4/8	>8/>8	NA/NA	40	0.04
*M. intracellulare*	40	4/2	4/2	>8/8	>8/>8	31	0.1
*M. chimaera*	35	2/1	4/2	8/8	>8/>8	29	0.15

*NA, not available, ECOFF above the test concentration range.*

Amikacin modal MICs were identical for the three species (*M. avium, M. intracellulare* and *M. chimaera*) in MH and 7H9 (16 mg/L) except for *M. intracellulare* in 7H9 (8 mg/L). Ethambutol, linezolid, moxifloxacin and rifabutin modal MICs were within one or two titer steps of each other for the three species in both media. Clarithromycin and rifampin modal MICs were higher for *M. avium* than for *M. intracellulare* and *M. chimaera*.

Three strains were unusual MAC members: one *M. timonense* and two *M. yongonense* for which it was not possible to draw any conclusion concerning the distribution of MIC values because of the limited sample size (Supplementary Table S3).

### Impact of the pH of the Medium on the MICs of Antibiotics Recommended for Treatment of MAC Infections

For MAC, modal MICs were the same in both media for amikacin (16 mg/L), linezolid (32 mg/L) moxifloxacin (2 mg/L) and rifabutin (≤0.25 mg/L), but not for rifampin (2 mg/L in MH vs. > 8 mg/L in 7H9), clarithromycin (2 mg/L in MH vs. 8 mg/L in 7H9) and ethambutol (4 mg/L in MH vs. 8 mg/L in 7H9) (Table 2).

The concordance percentages for MIC values determined in both media are low to moderate: from 27% for clarithromycin to 60% for linezolid. These results are confirmed by the kappa test revealing a very weak agreement for clarithromycin, ethambutol, rifabutin and rifampin (0 < k ≤ 0.2), a weak agreement for amikacin and moxifloxacin (0.2 < k ≤ 0.4) and a moderate agreement for linezolid (0.4 < k ≤ 0.6) (Table 2).

When MICs of the four drugs whose breakpoints were available (Table 1) were converted into interpretive categories, the kappa test showed a weak agreement for amikacin and moxifloxacin, a moderate agreement for linezolid and an excellent agreement for clarithromycin (k ≥ 0.8). The concordance percentage was between 59% (moxifloxacin) and 98% (clarithromycin) (Table 3). VME and ME were observed for moxifloxacin (respectively, 3 and 2%) and linezolid (2% for both), and mE were observed for all drugs, up to 36% for moxifloxacin (Table 3).

**TABLE 3:** Comparison of amikacin, clarithromycin, linezolid and moxifloxacin susceptibility results determined using SLOMYCO Sensititre^TM^ panels (TREK Diagnostic Systems) according to the medium employed (very major errors are given in bold and underlined, major errors in bold, and minor errors are underlined).

			**7H9**			**% Agreement (kappa)**
			**S**	**I**	**R**	
**Amikacin**	**MH**	**S**	132	2	0	87 (0.35)
		**I**	17	4	0	
		**R**	0	1	2	
**Clarithromycin**	**MH**	**S**	136	2	0	98 (0.92)
		**I**	1	1	0	
		**R**	0	0	18	
**Linezolid**	**MH**	**S**	14	7	**3**	73 (0.52)
		**I**	1	27	18	
		**R**	**3**	11	74	
**Moxifloxacin**	**MH**	**S**	27	5	**3**	59 (0.38)
		**I**	21	25	6	
		**R**	**5**	25	41	

Among strains resistant to clarithromycin or amikacin in the two media for which search for resistance mutations was performed, the results of the genotypic drug susceptibility testing were the following:

– among the 18 strains categorized resistant to clarithromycin in both media, 12 strains (67%) harbored an *rrl* mutation in at position 2058 or 2059,
– among the 2 strains categorized resistant to amikacin in both media, 1 harbored a mutation in *rrs* at the 1408 position.

Interestingly, among the strains categorized intermediate to clarithromycin or amikacin whatever the medium, no resistance mutation was found.

## Discussion

There are several issues concerning DST of NTM, from the lack of data regarding breakpoint availability to the scarcity of data establishing a correlation between *in vitro* assays and patient outcomes.

Recent studies have shown the impact of the media used on the determination of MICs for mycobacteria (Lavollay et al., 2014; Aziz et al., 2017). The goal of this study was to compare those measured in MH and 7H9 broths.

Regarding the reproducibility of results obtained with the reference strain *M. avium* ATCC 700898, we identified a major issue linked to the low percentage of agreement obtained with clarithromycin tested in MH broth (67%) whereas it was 100% in 7H9 broth. It could be related to antibiotic instability, a problem possibly influencing MIC measurements, as pointed out recently (Schoutrop et al., 2018). Indeed, this latter study revealed an important decrease of ≥75% in clarithromycin concentration in cation-adjusted MH broth medium over 14 days of incubation at 37°C while amikacin levels remain stable. In consequence, current MIC determinations could lead to false high MICs thus underestimating drug susceptibility.

CLSI and BTS recommend the performance of clarithromycin susceptibility testing of isolates taken prior to the initiation of treatment. As expected, the main difference between the MICs determined in the two media was observed for clarithromycin due to the difference in their pH (6.8 in 7H9 vs. 7.4 in MH) (Table 2 and Supplementary Figure S1; Truffot-Pernot et al., 1991). However, using previous CLSI breakpoints given for testing in 7H9 medium with radiometric testing (Clinical and Laboratory Standards Institute [CLSI], 2011), only rare minor errors were observed when MICs were converted into the SIR categories (2%) (Table 3).

Both guidelines also recommend the performance of amikacin susceptibility testing of isolates taken prior to initiation of treatment (Haworth et al., 2017; Clinical and Laboratory Standards Institute [CLSI], 2018). In both media, the MICs of amikacin were better correlated than those of clarithromycin (57 vs. 27%), but SIR concordance was lower (87 vs. 98%) (Tables 2, 3). In particular, when MICs were converted into SIR categories, 17 strains (11%) were categorized as I in MH and S in 7H9. Among these 17 strains, none was identified in patients who had been treated previously with amikacin. Moreover, of these, 12 were screened for mutation in *rrl*, none was mutated. Finally, the MIC distribution was Gaussian in MH and 7H9 media (Figure 1 and Supplementary Figures S1, S2). Thus, we believe that these 17 strains belong to the wild-type MAC strain population and should not be categorized as I. We propose to either modify the breakpoint in MH from 16 to 32 mg/L, or to measure the amikacin MIC in 7H9.

Susceptibility testing of moxifloxacin and linezolid may be considered for macrolide-resistant strains, although no *in vitro-in vivo* correlation has yet been established (Koh et al., 2013; Haworth et al., 2017; Clinical and Laboratory Standards Institute [CLSI], 2018). For linezolid and moxifloxacin, agreement was weak to moderate between both methods (Table 3), and in up to 5% of MICs that were converted into SIR categories there were VME and ME. Several reasons can explain this lack of agreement between the results obtained using the two media. The first is that the proposed breakpoints are spread through the distribution of the MICs of the wild-type strains (Figure 1 and Supplementary Figures S1, S2). Therefore, small MIC variations that are not clinically relevant may modify the categorization. The second reason is linked to the TREK microplate system. Reading is difficult, requires trained staff and is easier when the test is performed in 7H9 rather than in MH broth (personal observation). Overall, as MICs are high and there is no clear demonstration of activity of these drugs in humans (Schön and Chryssanthou, 2017), we propose not to perform *in vitro* susceptibility testing for moxifloxacin and linezolid.

Finally, although ethambutol and rifamycins are used for the treatment of MAC infections, no clinical breakpoints have been defined for these drugs (Clinical and Laboratory Standards Institute [CLSI], 2018). In their absence, agreement was measured based on MICs. It must be mentioned that for rifabutin and rifampin, the concentration range tested in broth does not cover the entire MIC distribution, either because concentrations are too high (rifabutin) or too low (rifampin) (Figure 1). However, despite overall high *in vitro* MICs, it has been shown that rifamycins and ethambutol do prevent selection of clarithromycin resistance (May et al., 1997; Gordin et al., 1999; Benson et al., 2003), and a recent study reported a better clinical response when rifampin and/or ethambutol MICs were below 8 mg/L (Kwon et al., 2018). If these results are confirmed, this concentration could be used as a clinical breakpoint.

Regarding MIC profiles among MAC members, our data confirm previous studies showing that MICs for *M. avium* are rather equivalent to or higher than those for other members of the MAC (Renvoisé et al., 2014; Litvinov et al., 2018). Also, rifampin MICs for *M. chimaera* isolates were lower than for *M. avium*, in line with a recent study (Maurer et al., 2018). Regarding the rarely described *M. timonense* and *M. yongonense*, our data are in favor of lower amikacin MICs for these species than for other members of MAC. Given the small number of strains in our study and in the literature, more data are needed to know the wild-type susceptibility profiles of these two species.

ECOFF values were close to those described in 2018 by Maurer et al., for clarithromycin, amikacin and rifabutin (a difference of no more than one dilution, except for rifabutin, in *M. chimaera*) and added data for ethambutol, rifampin and moxifloxacin (Maurer et al., 2018).

In conclusion, there is no major difference between MICs measured in 7H9 and MH broth for most antibiotics with the exception of clarithromycin and amikacin. For clarithromycin, the MIC values obtained with the two media evaluated are significantly different. Nevertheless, there is no clinical impact on SIR categorization when using breakpoints adapted to the media employed. However, for amikacin, 7H9 broth allows a better distinction between wild and resistant populations by limiting the number of strains categorized “intermediate.” Moreover, the 7H9 broth seems to offer a better growth of mycobacteria than the MH medium which makes it possible to render the results of the DST faster (from the first reading on day seven) without requiring a second reading on D10 or D14 as it can happen with the MH broth.

In addition, better growth of mycobacteria facilitates the reading of MICs, thus reducing inter-operator variability and improving the reproducibility of the technique. Since the only difference we’ve shown between the two media are in favor of the 7H9 medium, we recommend using the 7H9 broth supplemented with 5% OADC instead of CAMH broth supplemented with 5% OADC for DST of MAC.

## Members of the CNR-MyRMA

Members of the CNR-MyRMA are Emmanuelle Cambau, Faïza Mougari, Emmanuel Lécorché, Vincent Jarlier, and Isabelle Bonnet.

## Data Availability Statement

All datasets generated for this study are included in the article/Supplementary Material.

## Ethics Statement

According to French law at the time of start of the study and in accordance with the ethical standards of our hospitals’ institutional review boards (Committee for the Protection of Human Subjects), informed consent was not obtained because this observational study did not modify existing diagnostic or therapeutic strategies.

## Author Contributions

NV and AA conceived and designed the study. JJ, AA, FM, FB, WS, TM, and NV performed the experiments and collected the data. JJ wrote the manuscript which was reviewed and approved by AA, FM, FB, JR, WS, TM, and NV.

## Conflict of Interest

The authors declare that the research was conducted in the absence of any commercial or financial relationships that could be construed as a potential conflict of interest.

## Abbreviations

BTS: British Thoracic Society
CLSI: Clinical and Laboratory Standards Institute
DST: drug susceptibility testing
I: intermediate susceptibility
MAC: *Mycobacterium avium* complex
ME: major error
mE: minor error
MH: Mueller Hinton
MIC: minimum inhibitory concentration
NTM: nontuberculous mycobacteria
R: resistance
S: susceptibility
SGM: slowly growing mycobacteria
VME: very major error.
