# Supplementary material for: Rational Choice of Antibiotics and Media for Mycobacterium avium Complex Drug Susceptibility Testing
Source: Front Microbiol. 2020 Feb 19;11:81. doi: 10.3389/fmicb.2020.00081 (PMC7042387; doi:10.3389/fmicb.2020.00081)
Supplement: Supplementary file 1 [file Data_Sheet_1.docx]

**Supplementary material:**

**Table S1: Range of concentrations tested for the seven antibiotics used in this study**

| **Antimicrobial agent** | **Range of concentrations tested (mg/L)** |
| --- | --- |
| Amikacin | 1 - 64 |
| Clarithromycin | 0.06 - 64 |
| Ethambutol | 0.5 - 16 |
| Linezolid | 1 - 64 |
| Moxifloxacin | 0.12 - 8 |
| Rifabutin | 0.25 - 8 |
| Rifampin | 0.12 - 8 |

**Table S2: Reproducibility results interpreted with reference values provided by the CLSI**

| Antibiotic | Medium | Number of results with a concentration difference of one log_2_ or more compared to the reference values | | | | | Reference values for  *M. avium* ATCC 700898 | % Agreement |
| --- | --- | --- | --- | --- | --- | --- | --- | --- |
|  |  | **≥ -2** | **-1** | **0** | **1** | **≥ 2** |  |  |
| Clarithromycin | MH | 0 | 2 | 4 | 0 | 0 | 0.5-2 | 67 |
|  | 7H9 | 0 | 0 | 6 | 0 | 0 | 1-4 | 100 |
| Linezolid | MH | 0 | 0 | 6 | 0 | 0 | 4-16 | 100 |
|  | 7H9 | 0 | 0 | 6 | 0 | 0 | nd | 100 |
| Moxifloxacin | MH | 0 | 0 | 6 | 0 | 0 | 0.25-2 | 100 |
|  | 7H9 | 0 | 0 | 6 | 0 | 0 | nd | 100 |

**nd : not determined**

**Table S3: MICs for three unusual strains of the *M. avium* complex (1 *M. timonense* and 2 *M. yongonense*) compared to the modal MIC of the *M. avium* complex**

|  | **MIC (MH/7H9) mg/L** | | | | | | |
| --- | --- | --- | --- | --- | --- | --- | --- |
|  | **Amikacin** | **Clarithromycin** | **Ethambutol** | **Linezolid** | **Moxifloxacin** | **Rifabutin** | **Rifampin** |
| ***M. timonense*** | 4/4 | 0.5/2 | 1/4 | 8/16 | 2/2 | <0.25/<0.25 | 2/2 |
| ***M. yongonense*** | 8/8 | 1/1 | 4/2 | 32/32 | 1/1 | <0.25/0.5 | 2/8 |
| ***M. yongonense*** | 8/8 | 4/2 | 4/4 | 64/16 | 2/4 | 1/0.5 | 4/4 |
| ***M. avium* complex modal MIC** | 16/16 | 2/8 | 4/8 | 32/32 | 2/2 | ≤0.25/≤0.25 | 2/>8 |

**Figure S1: Distribution of MICs for *M. avium, M. intracellulare* and *M. chimaera* clinical isolates determined in MH medium (vertical bars represent the CLSI susceptibility breakpoints)**

Breakpoints for amikacin IV are represented in full lines and for liposomal or inhaled amikacin in a dotted line

**Figure S2: Distribution of MICs for *M. avium, M. intracellulare* and *M. chimaera* clinical isolates determined in 7H9 medium (vertical bars represent the CLSI susceptibility breakpoints)**

Breakpoints for amikacin IV are represented in full lines and for liposomal or inhaled amikacin in a dotted line
